# Supplementary material for: A vehicle license plate data access model based on the jump hash consistency algorithm
Source: PLoS One. 2023 Aug 24;18(8):e0288427. doi: 10.1371/journal.pone.0288427 (PMC10449116; doi:10.1371/journal.pone.0288427)
Supplement: Attachment — Submitted filename: Response to Reviewers.docx [file pone.0288427.s002.docx]

**Response to reviewer1:**

**Question** 1:  In “Algorithm 1”, is the “Algorithm 1” correct? Operator symbol “>>>” ?

**Answer**: Yes, it is right. “>>>” means an unsigned right shift. The experimental results indicate that there is no problem here.

**Question** 2: In Figure 8, the data label “Hbase” should be “HBase”.

**Answer**: Thank you. I have revised it.

**Question** 3: The abbreviation format of nouns in the text is not uniform, for example: linear congruence method (LCG), jump consistent hash algorithm (JCHA).

**Answer**: Thank you. I have carefully checked to ensure consistency in naming conventions.

**Question** 4: You’d better figure out the exact uniform variance of the data distribution in your experiment, and list the corresponding variance criteria.

**Answer**: Thanks for your suggestion. I give the variance of the data distribution in my experiment by table 2.

**Question** 5: The experimental part should increase the comparison with traditional methods in performance and characteristics, showing advantages and breakthroughs.

**Answer**: I Added section 5.4 in the manuscript. In this section, a qualitative comparison analysis was conducted with traditional data access models from the perspectives of whether it supports multi conditional queries, disk usage, memory consumption, horizontal scalability, and whether it supports real-time access.

**Question** 6: It is suggested to increase your comparison model, especially the model in recent years, which can increase persuasion of the paper’s method.

**Answer**: Added qualitative comparison analysis to the models in recent years, such as based on auxiliary index technology models, based on MapReduce technology models, and based on Spark technology models in the article.

**Question** 7: Some important references are missed. The authors should analyze more new related works. E.g. A Federated Learning-based License Plate Recognition Scheme for 5G-enabled Internet of Vehicles, IEEE Transactions on Industrial Informatics 17, no.(12) , pp.8523–8530, 2021

**Answer**: Thanks for you suggestion. I added references to the latest research findings.

**Response to reviewer2:**

**Question** 1: Paper is well written but overall proofreading is required to avoid minor spelling/typing mistakes.

**Answer**: Thanks for your careful review. I have carefully checked and corrected the spelling/typing mistakes.

**Question** 2: Authors should justify the novelty of their work.

**Answer**: In the section of result, I emphasized the novelty of this work. It includes: supporting multi-condition query fully, saving storage space, low memory usage, efficiency, scalability, and can meet the needs of real-time data access.

**Question** 3: Results should be compared with some state of the art technique.

**Answer**: I Added section 5.4 in the manuscript. In this section, a qualitative comparison analysis was conducted with traditional data access models from the perspectives of whether it supports multi conditional queries, disk usage, memory consumption, horizontal scalability, and whether it supports real-time access.

**Response to reviewer3:**

**Question 1**. In the introduction section, the current status and development trends of intelligent transportation systems can be further introduced, and the application and value of license plate data generated by intelligent transportation systems can be described in more detail.

**Answer**: Thanks for your suggestions. I have added content about trends in ITS and some detailed information about the value of license plate data.

**Question 2:** In the experimental section, more detailed experimental details and parameter settings can be provided so that readers can replicate the experiment and verify the results. Verification of the correctness of query results can be provided, for example, by comparing query results with manually counted results or query results from other databases.

**Answer**: I listed detail result data in table 2 and added a note in this paper about the consistency of the experimental results. Thank you.

**Question 3:** In the conclusion, the limitations of the algorithm can be added, and future research directions can be discussed. For example, the algorithm model has efficiency and scalability, and can meet the needs of real-time data access. In future research, we can explore other types of traffic data processing algorithms and further optimize the algorithm model proposed in this paper to meet the needs of different application scenarios.

**Answer**: Thanks for your great suggestion. The conclusion section is further refined by adding the strengths and limitations of the algorithms described in this paper and directions for future research.
